# Goreisan May Reduce Postoperative Swelling and Pain After Total Hip Arthroplasty: A Retrospective Comparative Study

**DOI:** 10.3390/jcm15062317

**Published:** 2026-03-18

**Authors:** Yuto Uehara, Norio Imai, Yasuhito Takahashi, Yuki Endo, Keishi Kimura, Yuki Hirano, Yoji Horigome, Hiroyuki Kawashima

**Affiliations:** 1 Department of Regenerative and Transplant Medicine, Division of Orthopedic Surgery, Niigata University Graduate School of Medical and Dental Sciences, Niigata 951-8510, Japan; 2 Division of Comprehensive Musculoskeletal Medicine, Niigata University Graduate School of Medical and Dental Sciences, Niigata 951-8510, Japan; 3 Department of Orthopedic Surgery, Niigata Rosai Hospital, Niigata 942-8502, Japan

**Keywords:** Goreisan, edema, total hip arthroplasty, postoperative pain, thigh circumference, Kampo medicine

## Abstract

**Background**: To our knowledge, the effect of Goreisan on edema following total hip arthroplasty (THA) has not been investigated, and its clinical efficacy in this context remains unclear. **Methods**: To elucidate these issues, we conducted a retrospective comparative study of 149 patients who underwent primary THA at our institutions, with group allocation based on treatment period. Patients were divided into control (*n* = 77, mean ± standard deviation age: 66.8 ± 9.8 years; 56 females) and Goreisan (*n* = 72, age: 65.6 ± 9.7 years; 53 females) groups, based on whether Goreisan was administered postoperatively for 7 days (2.5 g, three times daily). The primary outcome was CT-based postoperative swelling, assessed by thigh circumference and cross-sectional area, whereas pain and blood loss were evaluated as secondary outcomes. Correlation and multiple regression analyses were also conducted. **Results**: The postoperative blood loss was lower in the Goreisan group (451.2 ± 256.8 mL vs. 553.6 ± 301.9 mL, *p* = 0.036). The differences in thigh circumference and area were larger in the control group (+13.5 ± 12.6 mm vs. +1.9 ± 12.7 mm, *p* < 0.001; +11.1 ± 9.5 cm^2^ vs. +2.7 ± 9.3 cm^2^, *p* < 0.001). The enlargement ratios of thigh circumference and area were higher in the control group (+3.0 ± 2.96% vs. +0.41 ± 2.86%, *p* < 0.001; +6.96 ± 6.95% vs. +1.63 ± 5.70%, *p* < 0.001). Motion pain on postoperative day 7 was lower in the Goreisan group (1.71 vs. 2.18, *p* = 0.037). The differences and enlargement ratios of both the thigh circumference and area were associated with motion pain on postoperative days 1 and 7. In both cases, the correlations were stronger for circumference-related parameters than those for area-related parameters. **Conclusions**: These findings suggest that Goreisan may be associated with reduced postoperative swelling and pain after THA, although the results are observational and hypothesis-generating.

## 1. Introduction

Postoperative pain is a substantial concern for patients who undergo total hip arthroplasty (THA), as it may impede early mobilization, prolong hospitalization, and increase healthcare costs [1,2,3]. Among the factors contributing to postoperative pain, local swelling around the surgical site reportedly increases the tissue pressure and irritates the peripheral nervous system through both direct mechanical compression and the release of inflammatory cytokines [4,5,6,7]. Postoperative bleeding is considered a primary cause of such swelling and subsequent pain. Therefore, tranexamic acid, an antifibrinolytic agent, is often administered perioperatively to reduce surgical site bleeding, aiming to minimize swelling and alleviate pain [8,9,10,11].

Few reports on swelling and pain after THA in patients receiving Saireito have been published. Kishida et al. [6] reported that such patients exhibited reductions in thigh swelling and serum C-reactive protein levels compared with controls. Saireito—composed of Goreisan and Sho-saiko-to—has been associated with rare but serious adverse effects such as interstitial pneumonia [12,13,14] and severe hepatic dysfunction [15,16].

Goreisan (*Alisma orientale* (Sam.) Juz., *Poria cocos* (Schw.) Wolf, *Atractylodes lancea* (Thunb.) DC., *Polyporus umbellatus* (Pers.) Fr., and *Cinnamomum cassia* (L.) J. Presl), a traditional Kampo medicine originating from classical Chinese medicine and widely adopted in Japan, has long been prescribed for the treatment of edema, thirst, and dysuria in traditional medical practice [17]. Traditionally, it is believed to regulate body fluid balance and relieve swelling. Goreisan reportedly regulates body fluid distribution by inhibiting aquaporins 2, 3, and 4, thereby reducing water permeability into tissues [18,19,20,21,22]. This mechanism has been associated with the alleviation of cerebral edema [22] and treatment-resistant congestive heart failure [18]. Based on its fluid-modulating properties, we hypothesized that Goreisan may reduce edema, and consequently, alleviate pain following THA. To our knowledge, however, the effect of Goreisan specifically on postoperative edema and pain after THA has not been studied, and its clinical efficacy in this context remains unclear. In addition, postoperative swelling after THA is multifactorial and differs pathophysiologically from systemic or cerebral edema, involving surgical trauma, bleeding, and inflammatory responses. Therefore, it cannot be assumed that the fluid-modulating effects of Goreisan observed in other conditions would necessarily translate into reduced postoperative swelling in orthopedic surgery. Therefore, the purpose of this study was to investigate whether the standardized administration of Goreisan affects postoperative swelling and pain in patients who undergo THA. We hypothesized that postoperative Goreisan administration would be associated with reduced postoperative thigh swelling and lower pain scores after primary THA.

## 2. Methods

### 2.1. Participants

**Figure 1:**
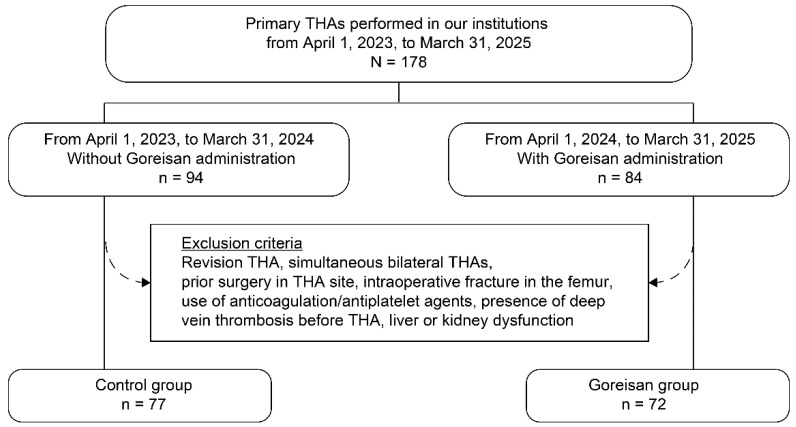
Flowchart of patient selection and group allocation. Among patients who underwent primary THA at our institutions from April 2023 to March 2025, those treated from April 2023 to March 2024 were assigned to the control group (no Goreisan administration), and those treated from April 2024 to March 2025 were assigned to the Goreisan group. THA, total hip arthroplasty.

Among patients who underwent primary THA at Niigata University Graduate School of Medical and Dental Science and Niigata Rosai Hospital from April 2023 to March 2025, those treated from April 2023 to March 2024 were assigned to the control group (no Goreisan administration), and those treated from April 2024 to March 2025 were assigned to the Goreisan group (Figure 1). For this retrospective review, patients were excluded if they underwent revision surgery, underwent simultaneous bilateral THA, or had undergone previous hip surgery. Patients with liver or kidney dysfunction were also excluded. In addition, patients receiving anticoagulants or antiplatelet agents and those with intraoperative fractures were excluded, as these factors may influence perioperative bleeding. Finally, we excluded patients with deep vein thrombosis (DVT) identified via preoperative lower extremity venous ultrasound.

### 2.2. Study Protocol

This study was a retrospective non-randomized comparative cohort study, with group allocation based on treatment period. The primary outcome of this study was postoperative swelling, assessed by the circumference enlargement ratio on postoperative day 7. Secondary outcomes included postoperative pain scores, postoperative blood loss, and the incidence of deep vein thrombosis and liver dysfunction.

Preoperative blood tests, CT imaging, and lower-extremity ultrasonography were routinely performed approximately one month before surgery as part of the institutional preoperative assessment protocol. The preoperative NRS score was evaluated after hospital admission before surgery.

The evaluated demographic and clinical parameters were age, sex, right or left hip, and body mass index (BMI). Blood tests to determine the hemoglobin (Hb) level, hematocrit (Hct), platelet count (Plt), activated partial thromboplastin time (aPTT), prothrombin time (PT), aspartate aminotransferase (AST) concentration, alanine aminotransferase (ALT) concentration, and D-dimer (D-D) concentration were performed preoperatively and on postoperative days 1, 3, and 7. In addition, intraoperative blood loss was recorded, and the estimated actual blood loss (eABL) was calculated using the following formulae, proposed by Gross [23]:

eABL (mL) for men = 70 × body weight × ([Hct of day 7 − Hct before surgery]/mean of Hct before surgery and day 7)

and

eABL (mL) for women = 65 × body weight × ([Hct of day 7 − Hct before surgery]/mean of Hct before surgery and day 7).

**Figure 2:**
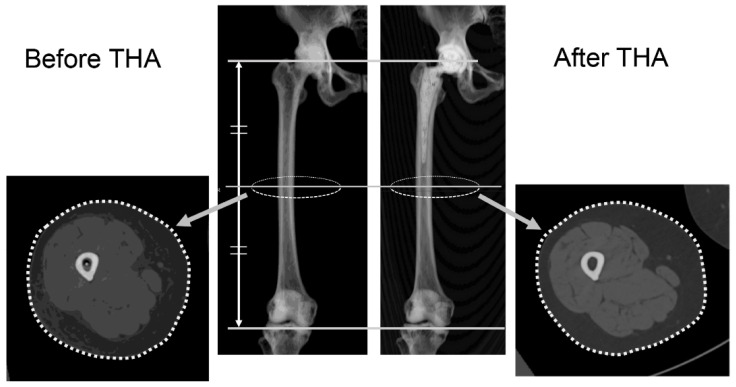
Measurement of thigh circumference and cross-sectional area using CT images before and after THA. After the femur was aligned to the radiographic coronal plane, the circumference and cross-sectional area of the thigh were measured on a plane perpendicular to the *z*-axis, which passes through the midpoint between the tip of the greater trochanter and the most distal end of the femoral condyles. CT, computed tomography; THA, total hip arthroplasty.

We calculated blood loss attributed only to the postoperative period by subtracting the intraoperative blood loss from the eABL. With regard to AST and ALT, when their concentrations exceeded thrice the normal upper limit according to the respective institution’s standards (for example, if the normal upper limit was 35 U/L and the measured concentrations were above 105 U/L), we diagnosed a patient with liver dysfunction [24], a common adverse event related to Goreisan. The thigh circumference and cross-sectional area were measured using computed tomography (CT) images obtained on postoperative day 7, originally acquired for postoperative verification of the implant position and alignment [25,26,27]. Measurements were performed using ZedView software (version 18.0.0; LEXI Co., Ltd., Tokyo, Japan) [28,29]. After the femur was aligned to the radiographic coronal plane [30], the circumference and cross-sectional area of the thigh were measured on a plane perpendicular to the *z*-axis, which passes through the midpoint between the tip of the greater trochanter and the most distal end of the femoral condyles (Figure 2).

**Figure 3:**
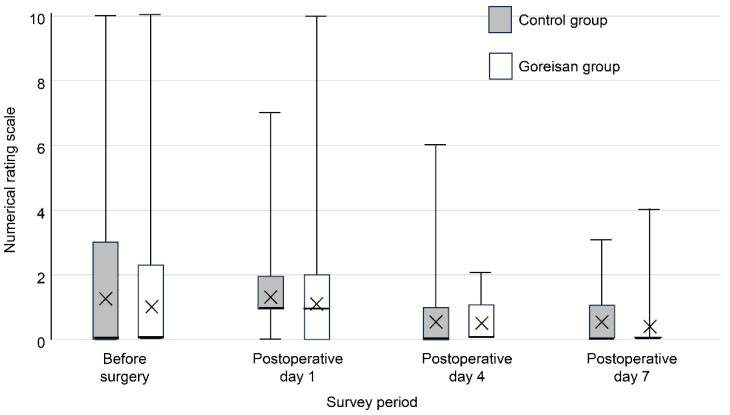
Resting pain score before and after THA. Although the resting NRS scores were lower in the Goreisan group, the difference was not statistically significant. NRS, numerical rating scale; THA, total hip arthroplasty.

**Figure 4:**
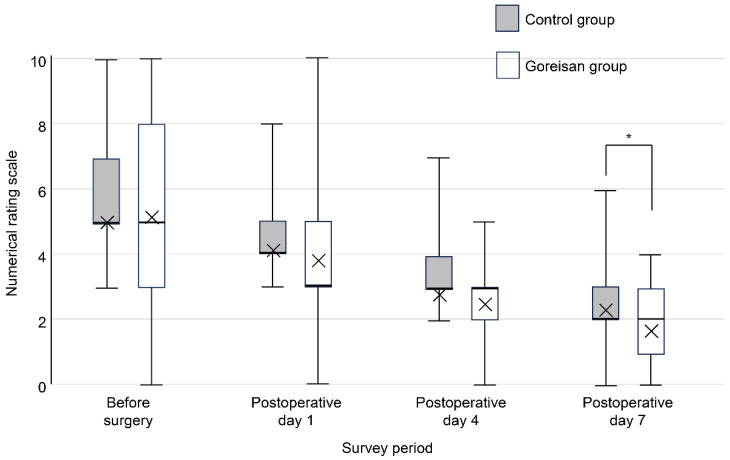
Motion pain score before and after THA. Regarding perioperative pain assessed using the NRS, motion pain on postoperative day 7 was lower in the Goreisan group (* *p* = 0.037). THA, total hip arthroplasty.

Perioperative pain was assessed (both at rest and during motion) using an 11-point numerical rating scale (NRS), ranging from 0 (no pain) to 10 (the worst imaginable pain). For the assessment of motion-related pain, the NRS was recorded during the transition from the sitting to the standing position, which was standardized for all patients. Pain levels were evaluated preoperatively and on postoperative days 1, 4, and 7 (Figure 3 and Figure 4). On postoperative day 7, the presence or absence of DVT was evaluated using lower-extremity ultrasonography.

### 2.3. Materials

In this study, we used a commercially available formulation of Goreisan (TJ-17; Tsumura & Co., Tokyo, Japan). The specific batch (lot) number was not available. According to the manufacturer’s instructions, the product complies with established quality control standards and specifications. Information regarding the quantitative analysis of known active compounds is based on the manufacturer’s data.

All THAs were performed by experienced orthopedic surgeons under general anesthesia via the anterolateral supine (ALS) approach [31,32,33] according to the standardized institutional protocol. To prevent bleeding, 1 g of tranexamic acid was administered intravenously prior to surgery. For thromboprophylaxis, a foot pump was used intraoperatively, and compression stockings were worn postoperatively until discharge. In the Goreisan group, Goreisan was administered at a dose of 2.5 g, three times daily for 7 days, starting at lunch time on the first postoperative day. In Japan, the standard adult dosage of Goreisan (TJ-17; Tsumura & Co.) is 2.5 g three times daily according to the approved prescribing information. Therefore, all patients in the Goreisan group received the same fixed dose, and dose adjustment according to body weight or other patient characteristics was not performed. Full weight-bearing was permitted from the first postoperative day, and patients were typically discharged home approximately 10 days after surgery according to the standard postoperative protocol.

### 2.4. Statistical Analysis

Because this study was designed as a retrospective observational study using all eligible patients during the study period, an a priori sample size calculation was not performed. Instead, a post hoc power analysis was conducted to assess the statistical validity of the study.

Statistical analyses were performed using IBM SPSS Statistics for Windows (ver. 28; IBM Corp., Armonk, NY, USA). Quantitative variables (age, BMI, Hb level, Hct, Plt, aPTT, PT, AST concentration, ALT concentration, D-D concentration, intraoperative blood loss, eABL, postoperative blood loss, thigh circumference, and cross-sectional area) were compared between the control and Goreisan groups by using the unpaired *t*-test. For the nonparametric variable, the NRS score, the Mann–Whitney U test was applied. For qualitative variables (sex, laterality, and the presence of DVT), the chi-square test or Fisher’s exact test was applied, as appropriate.

Pearson’s correlation coefficient was used to identify factors associated with the circumference difference, circumference enlargement ratio, area difference, and area enlargement ratio. Spearman’s rank correlation coefficient was used to identify factors associated with the perioperative NRS score. We visualized NRS scores as a boxplot with medians and quartiles. Variables that showed significant associations in the univariable analyses were included in the multivariable regression model. Furthermore, multivariable linear regression analysis was performed using either rest or motion pain—whichever was significantly correlated with other variables in Spearman’s rank analysis; if both were correlated, motion pain was used—as the dependent variable and the other relevant parameters as independent variables.

For thigh circumference and cross-sectional area measurements, the same examiner measured each parameter twice at an interval of more than 1 week to evaluate intra-observer reliability, and two independent orthopedic surgeons performed separate measurements to assess inter-observer reliability. We also performed a post hoc analysis to evaluate statistical power (type II [β] error). We defined the effect size (d) as 0.5 and type I (α) error as 0.05 for the *t*-test, and we defined d as 0.3 and α as 0.05 for the chi-squared test. Intraclass correlation coefficients (ICCs) were calculated for both intra- and inter-observer reliability. A two-tailed *p*-value < 0.05 was considered statistically significant.

### 2.5. Ethical Considerations

This study was conducted in accordance with ethical standards for research involving human subjects and the principles of the Declaration of Helsinki. The study protocol was approved by the Institutional Review Board of Niigata University (approval number: 2025-0056). Because this was a retrospective observational study, written informed consent was not required by the Board; instead, an opt-out approach was employed.

## 3. Results

The participants’ background characteristics (age, sex, BMI, and laterality) did not significantly differ between groups (Table 1). Although the groups also did not significantly differ in terms of operative time or intraoperative blood loss, postoperative blood loss was significantly lower in the Goreisan group (Table 1). The pre- and postoperative thigh circumference and cross-sectional area did not significantly differ between the groups. However, the differences in circumference and area (postoperative minus preoperative values), as well as the enlargement ratios (postoperative divided by preoperative values), were significantly larger in the control group (Table 2). Motion pain on postoperative day 7 was significantly lower in the Goreisan group (2.18 in the control group and 1.71 in the Goreisan group, *p* = 0.037; Figure 4).

According to Pearson’s correlation analysis, BMI was positively correlated with operative time, intraoperative blood loss, eABL, and postoperative blood loss but negatively correlated with the area enlargement ratio (Table 3). Preoperative thigh circumference and cross-sectional area were positively correlated with operative time, intraoperative blood loss, eABL, and postoperative blood loss, whereas they were negatively correlated with the circumference difference, area difference, and area enlargement ratio (Table 3).

According to Spearman’s correlation analysis, the circumference difference, area difference, circumference enlargement ratio, and area enlargement ratio were associated with motion pain on postoperative days 1 and 7. In both cases, the correlations were stronger for the circumference difference and enlargement ratio than for area-related parameters (Table 4).

Multiple linear regression analysis was performed using motion pain on postoperative day 1 as the dependent variable, based on the significant correlations identified via Spearman’s analysis. Only the circumference enlargement ratio was an independently associated factor (correlation coefficient: 0.281 [95% confidence interval (CI): 0.018–0.542], *p* = 0.039). The same was true when motion pain on postoperative day 7 was used as the dependent variable (correlation coefficient for the circumference enlargement ratio: 0.276 [95% CI: 0.210–0.342], *p* = 0.028).

The incidence of DVT was 8% (six cases) in the control group and 10% (seven cases) in the Goreisan group (*p* = 0.677). With regard to liver dysfunction, the incidence was 3% (two cases) in the control group and 3% (two cases) in the Goreisan group (*p* = 0.946).

Post hoc analysis revealed that the statistical power was 0.858 for the *t*-test, 0.949 for the Mann–Whitney U test, and 0.968 for correlation analysis. The intra-observer reliability was excellent, with ICCs of 0.999 for circumference measurements and 0.998 for area measurements. Inter-observer reliability was also high, with ICCs of 0.994 for both parameters.

## 4. Discussion

In the present study, the Goreisan group exhibited significantly smaller differences and enlargement ratios for both the circumference and area of the thigh on CT than the control group. These findings may reflect the fluid-regulating properties of Goreisan. Previous studies have suggested that Goreisan reduces edema by modulating water distribution at the tissue level, possibly through aquaporin-related mechanisms [18,19,20,21,22,34,35,36]. Although aquaporins were not directly evaluated in the present study, a similar mechanism may have contributed to the suppression of postoperative edema at the surgical site following THA.

Conventional diuretics act on the proximal and distal renal tubules, loop of Henle, and the collecting ducts, promoting the excretion of sodium ions via osmotic gradients, which in turn induces water excretion [37,38]. In contrast, Goreisan is known to suppress the function of aquaporins, exhibiting a water-selective diuretic effect—referred to as a “regulatory effect on body fluid” [39]. As a result, Goreisan may increase urine output without substantially affecting plasma electrolyte concentrations. Moreover, this diuretic effect appears only under edematous conditions and does not occur in dehydrated states, rendering Goreisan a unique therapeutic agent in terms of fluid regulation [40,41,42]. It is considered to have a lower risk of serious side effects [43].

Although operative conditions were similar between the two groups, Goreisan administration was associated with reduced postoperative blood loss and lower motion pain in the early postoperative period. These findings are consistent with previous reports suggesting possible anti-inflammatory and hemostatic effects of Goreisan. Previous studies have revealed the anti-inflammatory [44,45,46] and hemostatic [47,48] properties of Goreisan, which are consistent with the outcomes in this study. Furthermore, the incidence of DVT was not higher in the Goreisan group. This may suggest that Goreisan, similar to tranexamic acid, acts on the fibrinolytic system rather than the coagulation cascade.

Higher BMI was positively correlated with operative time, intraoperative blood loss, eABL, and postoperative blood loss, but negatively correlated with the area enlargement ratio. Similarly, greater preoperative thigh circumference and cross-sectional area were positively correlated with operative time, intraoperative blood loss, eABL, and postoperative blood loss, but negatively correlated with circumference difference, area difference, and area enlargement ratio. These findings suggest that patients with a lower BMI and smaller preoperative thigh circumference and cross-sectional area are less likely to experience significant perioperative blood loss but may be more prone to postoperative swelling.

Circumference difference, area difference, circumference enlargement ratio, and area enlargement ratio were all associated with motion pain on postoperative days 1 and 7. In both instances, the correlations were stronger for the circumference difference and enlargement ratio than for area-related measures. Furthermore, when motion pain on postoperative days 1 and 7 was used as the dependent variable in multiple regression analysis, only the circumference enlargement ratio emerged as a significant associated factor. These findings suggest that postoperative pain related to swelling is more strongly associated with changes in the thigh circumference than it is with changes in the thigh cross-sectional area. One possible explanation is that, during the early stages of swelling, the thigh cross-section may become more circular while maintaining the same circumference, and therefore may not substantially increase tissue pressure or pain. As swelling progresses, however, an increase in circumference may lead to elevated compartmental pressure, which in turn may contribute to pain [1,2,3]. Thus, early increases in cross-sectional area may have a limited impact on postoperative pain. Additionally, previous studies have suggested that neural edema may contribute to pain and that Goreisan may alleviate pain symptoms by reducing such edema [49]. In the present study, the analgesic effect of Goreisan might have been at least partially mediated by improvements in neural edema.

In previous studies, postoperative edema was typically assessed by measuring thigh circumference at fixed anatomical landmarks on the skin surface, using a measuring tape [6]. However, this method is susceptible to measurement error owing to variability in tape positioning and compression during measurement. Moreover, to ensure intra- and inter-observer reliability, multiple measurements—typically at least three—must be performed at the same time of day, which can be burdensome for both examiners and patients. In contrast, the method used in the present study was based on CT images obtained on postoperative day 7, which were originally acquired for three-dimensional postoperative evaluation of implant position and alignment. By aligning the femur to the same coordinate system before and after surgery, this technique offers excellent reproducibility and high reliability. To our knowledge, this is the first report to describe such a precise and reproducible method for quantifying postoperative thigh swelling. In our institutions, postoperative CT is routinely performed to confirm the position and alignment of the implant after THA [25,26,27]. Therefore, the CT images used in this study were not obtained specifically for the purpose of evaluating postoperative swelling but were retrospectively utilized for this analysis. Although CT involves radiation exposure, no additional imaging was performed for research purposes. We do not recommend CT imaging solely for the assessment of postoperative swelling.

One of the major adverse events associated with Goreisan is liver dysfunction [50,51]. In the present study, the incidence of liver dysfunction did not significantly differ between the control and Goreisan groups. Administration of analgesics and antibiotics after THA can also induce liver dysfunction [24,52,53,54]. According to previous studies, the incidence of liver dysfunction is approximately 1% even without Goreisan administration [50], which is consistent with the findings in both groups in the present study. Therefore, although no increase in liver dysfunction was observed in the Goreisan group, rare adverse events cannot be completely ruled out because of the limited sample size.

This study has several limitations. First, because the control and Goreisan groups were defined according to different time periods, temporal bias cannot be completely excluded. However, the perioperative management protocol, rehabilitation program, surgical approach, and implant selection remained essentially unchanged during the study period. In addition, this retrospective study was not blinded and did not adjust for potential center-related effects. Therefore, differences in perioperative management, including rehabilitation protocols or analgesic use, may also have influenced the results. Second, although it was a multicenter study, only approximately 150 patients were included. Because of the limited sample size, age and sex were not included in the multivariable model to avoid overfitting. In addition, although the underlying diagnoses leading to THA were reported, potential differences related to diagnosis or other anatomical factors were not adjusted for in the analysis. Therefore, residual confounding cannot be completely excluded. Nevertheless, post hoc analyses revealed power values greater than 0.8, indicating that the minimum required sample size to ensure the validity of the findings was achieved. Third, although significant correlations were observed, the correlation coefficients ranged from 0.2 to 0.3, indicating that the associations were weak. Although these correlation coefficients indicate modest associations, postoperative pain is inherently multifactorial and influenced by numerous biological and mechanical factors; therefore, small-to-moderate effect sizes are commonly observed in clinical orthopedic research. Furthermore, the CT-based measurements demonstrated excellent intra- and inter-observer reliability (ICCs ≥ 0.994), supporting the reproducibility of the morphological assessments. Nevertheless, due to the retrospective design and potential residual confounding, prospective randomized studies are warranted to confirm these findings. Fourth, all participants in this study were Japanese. Therefore, the generalizability of these findings to other populations and healthcare systems should be interpreted with caution. Fifth, length of hospital stay and functional outcome scores were not evaluated. In our institutions, discharge timing is often influenced by social factors such as family support and personal circumstances. Therefore, hospital stay may not accurately reflect postoperative swelling, pain, or functional recovery. In addition, swelling and pain were evaluated only during the early postoperative period. Additionally, all THAs in this study were performed using the ALS approach. This approach is considered less invasive than the posterior approach [31,32,33], and the choice of surgical approach may influence outcomes and should be considered when interpreting the results. Future studies including functional outcome measures such as the Harris Hip Score or HOOS and larger sample sizes are warranted to further validate these findings.

## 5. Conclusions

In this retrospective comparative study, postoperative Goreisan administration was associated with lower CT-based thigh swelling, reduced postoperative blood loss, and lower motion-related pain on postoperative day 7 after THA. Changes in thigh circumference were more strongly associated with postoperative motion pain than changes in cross-sectional area. Circumference-based swelling parameters were more closely associated with postoperative pain than area-based measurements. Prospective randomized studies are required to confirm these findings and clarify the underlying mechanisms.

## Figures and Tables

**Table 1:** Baseline cross-sectional demographic and clinical characteristics of the participants before surgery.

	Control Group (*n* = 77)	Goreisan Group (*n* = 72)	*p*-Value
Age (years) *	66.8 ± 9.8	65.6 ± 9.7	0.507 **
Sex (male/female)	21/56	19/53	0.903 ^†^
Right/left	35/42	32/40	0.887 ^†^
Primary disease			
Developmental dysplasia of the hip	62	60	0.890
Osteonecrosis of the femoral head	9	8	
Primary osteoarthritis	3	2	
Rapidly distractive arthritis	2	2	
Rheumatoid arthritis	1	0	
Body mass index (kg/m^2^) *	24.1 ± 4.2	22.5 ± 3.9	0.273 **
Hemoglobin (g/dL) *	12.8 ± 1.3	13.0 ± 1.1	0.550 **
Hematocrit (%) *	38.8 ± 3.3	39.4 ± 3.1	0.292 **
Platelets (10^4^/μL) *	24.2 ± 6.4	23.9 ± 4.7	0.318 **
Activated partial thromboplastin time (s) *	29.4 ± 3.5	28.4 ± 3.0	0.271 **
Prothrombin time (%) *	106.5 ± 24.0	112.0 ± 25.7	0.296 **
Prothrombin time-international normalized ratio *	0.96 ± 0.07	0.95 ± 0.10	0.298 **
Aspartate aminotransferase (U/L) *	32.2 ± 18.5	30.4 ± 17.7	0.385 **
Alanine aminotransferase (U/L) *	31.3 ± 24.6	30.7 ± 24.9	0.531 **
Surgical time (min) *	115.0 ± 26.5	114.1 ± 37.9	0.870 **
Intraoperative bleeding (mL) *	298.7 ± 164.4	314.0 ± 197.5	0.625 **
Estimated actual blood loss (mL) *	844.5 ± 374.7	757.4 ± 315.7	0.147 **
Postoperative blood loss (mL) *	553.6 ± 301.9	451.2 ± 256.8	0.036 **

* mean ± standard deviation, ** *t*-test, ^†^ chi-square test.

**Table 2:** Measurements related to thigh circumference and cross-sectional area.

	Control Group (*n* = 77)	Goreisan Group (*n* = 72)	*p*-Value
Circumference before THA (mm)	457.2 ± 50.9	464.4 ± 65.3	0.480 **
Circumference after THA (mm)	470.7 ± 49.8	466.3 ± 64.4	0.664 **
Area before THA (cm^2^)	159.5 ± 37.0	165.1 ± 48.5	0.457 **
Area after THA (cm^2^)	170.6 ± 37.0	167.8 ± 47.4	0.709 **
Difference in circumference before and after THA (mm)	+13.5 ± 12.6	+1.9 ± 12.7	<0.001 **
Enlargement ratio of circumference (%)	+3.0 ± 2.96	+0.41 ± 2.86	<0.001 **
Difference in area before and after THA (cm^2^)	+11.1 ± 9.5	+2.7 ± 9.3	<0.001 **
Enlargement ratio of area (%)	+6.96 ± 6.95	+1.63 ± 5.70	<0.001 **

Data are presented as the mean ± standard deviation. ** *t*-test.

**Table 3:** Pearson’s correlation.

	Body Mass Index	Surgical Time	Intraoperative Bleeding	Estimated Actual Blood Loss	Postoperative Blood Loss	Circumference Before Surgery	Area Before Surgery	Circumference After THA	Area After THA	Difference In Circumference	Difference In Area	Enlargement Ratio of Circumference	Enlargement Ratio of Area
Age							−0.215 (0.043)		−0.206 (0.047)				
Body mass index	-	0.335 (0.001)	0.284 (0.018)	0.394 (<0.001)	0.313 (0.007)	0.887 (<0.001)	0.889 (<0.001)	0.883 (<0.001)	0.889 (<0.001)				−0.216 (0.043)
Surgical time	0.335 (0.001)	-	0.552 (<0.001)	0.365 (<0.001)		0.243	0.254	0.275 (0.020)	0.283 (0.018)				
Intraoperative bleeding	0.284 (0.018)	0.552 (<0.001)	-	0.584 (<0.001)		0.216 (0.043)	0.211 (0.045)	0.275 (0.020)	0.272 (0.021)			0.214 (0.044)	
Estimated actual blood loss	0.394 (<0.001)	0.365 (<0.001)	0.584 (<0.001)	-	0.854 (<0.001)	0.335 (0.001)	0.333 (0.002)	0.399 (<0.001)	0.399 (<0.001)	0.206 (0.047)		0.234 (0.036)	
Postoperative blood loss	0.313 (0.007)			0.854 (<0.001)	-	0.288 (0.016)	0.287 (0.017)	0.328 (0.003)	0.325 (0.004)				
Circumference before surgery	0.887 (<0.001)	0.243 (0.030)	0.216 (0.043)	0.335 (0.001)	0.288 (0.016)	-	0.991 (<0.001)	0.972 (<0.001)	0.966 (<0.001)	−0.203 (0.048)	−0.237 (0.035)		−0.256 (0.026)
Area before surgery	0.889 (<0.001)	0.254 (0.026)	0.211 (0.045)	0.333 (0.002)	0.287 (0.017)	0.991 (<0.001)	-	0.964 (<0.001)	0.970 (<0.001)	−0.202 (0.049)	−0.221 (0.040)		−0.258 (0.024)

Upper low: correlation coefficient, lower low: *p*-value.

**Table 4:** Spearman’s correlation.

	Rest Pain Before Surgery	Motion Pain Before Surgery	Rest Pain Day 1	Motion Pain Day 1	Rest Pain Day 4	Motion Pain Day 4	Rest Pain Day 7	Motion Pain Day 7	Difference in Circumference	Difference In Area	Enlargement Ratio of Circumference	Enlargement Ratio of Area
Motion pain before surgery	0.369 (<0.001)	-										
Rest pain day 1	0.293 (0.015)		-	0.298 (0.012)	0.416 (<0.001)	0.326 (0.002)	0.301 (0.009)	0.326 (0.002)				
Motion pain day 1			0.298 (0.012)	-		0.539 (<0.001)		0.431 (<0.001)	0.237 (0.036)	0.216 (0.045)	0.248 (0.031)	0.210 (0.048)
Rest pain day 4	0.294 (0.014)	0.211 (0.048)	0.416 (<0.001)		-	0.451 (<0.001)	0.504 (<0.001)	0.285 (0.019)				
Motion pain day 4			0.326 (0.002)	0.539 (<0.001)	0.451 (<0.001)	-	0.256 (0.029)	0.658 (<0.001)				
Rest pain day 7	0.218 (0.044)		0.301 (0.009)		0.504 (<0.001)	0.256 (0.029)	-	0.353 (<0.001)				
Motion pain day 7			0.326 (0.002)	0.431 (<0.001)	0.285 (0.019)	0.658 (<0.001)	0.353 (<0.001)	-	0.262 (0.027)	0.212 (0.047)	0.254 (0.029)	0.215 (0.046)

Upper low: correlation coefficient, lower low: *p*-value.

## Data Availability

The datasets generated during and/or analysed during the current study are not publicly available due to ethical restrictions and the inclusion of personal medical information, but are available from the corresponding authors on reasonable request.
